# The effect of density on sex differentiation, sexual dimorphism, stress, and related gene expression in yellow perch

**DOI:** 10.1371/journal.pone.0267904

**Published:** 2022-05-04

**Authors:** Rafidah Othman, Han-Ping Wang, Hiam Elabd, Ding-Kun Xie, Hong Yao, Paul O’Bryant, Dean Rapp

**Affiliations:** Ohio Center for Aquaculture Research and Development, The Ohio State University South Centers, Piketon, OH, United States of America; University of Hyderabad, INDIA

## Abstract

A 180-day experiment was conducted to evaluate the effects of density on sex differentiation, sexual dimorphism, cortisol level, and stress related gene expression. Yellow perch, *Perca flavescens*, with initial mean body weight of 0.03 ± 0.001 g were reared in three different stocking densities: 1, 2, and 4 fish/L, termed as low (LD), moderate (MD), and high (HD) density, respectively, in a flow-through tank system. Results showed no significant differences in sex ratio in all density groups compared to normal population 1:1, and sexual size dimorphism (SSD) appeared when male and female were as small as the mean size reaching 11.5 cm and 12.3 cm in total length (TL) or 13.2g and 16.9g in body weight (BW), respectively. This female-biased sexual growth dimorphism was more pronounced in LD, although it was observed across all density groups. A significantly higher condition factor (K) of females than males in the LD group, and significantly higher R values of LD and MD than HD with the length/weight (L/W) linear relationships in females, were observed. Parallelly, fish reared in LD showed significantly higher mean body weight than those in the MD and HD groups, but there were no significant differences between the MD and HD. Similar results were also observed in all the other parameters of weight gain, specific growth rate (SGR), condition factor (K), and survival. These findings suggested that high density not only affected growth itself, but also affected SSD, growth trajectory or body shape, and general wellbeing in fish, especially in females. There were no significant differences in gonadosomatic index (GSI) and viscerosomatic index (VSI) among all the density groups; however, the hepatosomatic index (HSI) of LD was significantly higher than MD and HD, suggesting high density affected liver reserves or functions. Physiologically, plasma cortisol level was significantly highest in the LD among all groups, followed by MD, and lowest in HD. At the molecular level, the expression of the 70-kDa heat shock protein (*Hsp70)*, glutathione peroxidase (*GPx)*, and superoxide dismutase (*SOD)* genes involved in cellular stress were significantly upregulated in the HD group. The most significantly downregulated expression of these genes was consistently observed in the MD when compared to the LD and HD groups. In conclusion, increasing density induced chronic stress in yellow perch without affecting sex differentiation, but negatively affected expression of stress-related genes and mobilization of liver reserve, resulting in poorer wellbeing and reduced SSD, growth, and survival.

## Introduction

Yellow perch, *Perca flavescens*, has been known as an economically important fish species for recreational and commercial fisheries [1], and given top priority in research and Extension topics [2] in the North Central region of the United States. Intensive aquaculture is usually linked to stress and fish welfare. It is believed that environmental stress during development may influence phenotypic physiology and genetic factors [3, 4]. Fishes generally exhibit sexual plasticity for environmental effects on their reproduction [5–8], and sexual development is more plastic in non-mammalians, especially teleost fish (reviewed by Avise [9]). It has been reported that, in many species, the influence of temperature and other environmental factors on gonadal differentiation during the critical labile period is linked to the hyphothalamus-pituitary-internal axis and the main stress linked to corticosteroid cortisol [6, 10–12]. Stocking density in fish rearing has long been studied as a condition that could be deleterious to fish by disrupting their homeostasis and affecting immunocompetence [4, 13]. In eels, a previous study indicated that the density of individuals is the main factor affecting sex differentiation, with crowding favoring maleness [14]. The fish exhibit sexual size dimorphism (SSD), which may be associated with sex steroids [15–17]. Growth is also affected by sexual dimorphism in many species such as sea bass [18, 19], turbot [20], perch [21, 22], and walleye [23] with females growing faster than males. Previous studies reported that sexual dimorphism in yellow perch appears at 11.0 cm [24]. Stocking density is also a crucial variable with regard to growth performance, and normally inversely correlated in cultured fish [25], and differences in growth performance could be attributed to the onset of hierarchies and dominance relationship [26]. Hence, in intensive aquaculture, juvenile yellow perch are cannibalistic, which can cause significant losses during production [27]. Additionally, the establishment of hierarchies among fish under crowding conditions could lower food availability for subordinate fish and lead to anorexia [28] and increased energy expenditure [29]. Under stress conditions, animals’ response is characterized by the rapid release of stress hormones such as cortisol, resulting in the mobilization of energy reserves in an attempt to reestablish homeostasis [4, 30]. Apart from plasma cortisol level, the study of molecular biomarkers could be used for the assessment of fish stress and welfare conditions [4, 31]. As an example, heat shock protein 70 (*Hsp70*) is highly conserved and involved in various essential cellular processes in living cells as a molecular chaperone [4, 32]. Various studies have found that *Hsp70* expression is positively correlated with the level of stress [4, 33], where the synthesis can be triggered in response to several environmental and pathological stressors [4, 34]. *SOD* and *GPx* are among the first line of antioxidant defense in fish. Weight-length relationships in fish provide important information about general wellbeing, growth performance, health condition, fatness, habitat, and life history, as well as morphological criteria of the fish [35,36]. In fish farming management, it is very important to provide an ideal environment, especially during early development, as it can have a lasting effect on later life-history variation in fishes [37]. Hence, the present study has been carried out to also evaluate the gene expression of *Hsp70*, *SOD*, and *GPx* in liver, as it relates to stress and stocking density. Liver was chosen for being an important organ in the enzymatic transformation of ROS [38]. It was hypothesized that increasing stocking density would result in changes to those parameters. The main objective of this work was to study the influence of density on sex differentiation, growth performance, sexual growth dimorphism, and stress level in yellow perch juveniles. Population management of these juveniles at an optimal density is important to assure low mortality and better growth performance and wellbeing, especially during the grow-out phase, and also to be used as an approach for monosex production.

## Materials and methods

We confirmed that all the experiments and fish individuals involved were in accordance with the animal care and experimental procedures that were approved by the Institutional Animal Care and Use Committee of the Ohio State University.

### Fish and experimental design

The experimental fish were procured from the Aquaculture Research Center at The Ohio State University South Centers, Piketon, Ohio, USA. A total of 630 yellow perch at 35 dph with an average body weight (BW) of 0.03 ± 0.01 g and total length (TL) of 1.64 ± 0.19 cm were randomly stocked into 30L fiberglass experimental tanks. These fish were assigned into three experimental densities of low density (LD), medium density (MD), and high density (HD) of 30, 60, and 120 fish/tank or equal to 1,000, 2,000, and 4,000 fry per cubic meter, respectively. Three replicate tanks were stocked for each group for a total of nine experimental tanks. They were acclimated for one week and fed three times per day to apparent satiation with starter feed at the beginning and larger feed as they grew into juvenile a rate proportional to their density. All tanks had a constant flow-through water system and aeration, maintained at 23 ± 0.55°C and dissolved oxygen (DO) concentration at 6.0±0.65 mgL^-1^. Tanks were cleaned and water quality parameters were monitored on a daily basis throughout the experiment. All experimental procedures and methods involving animals in this study were approved and performed according to The Ohio State University’s Institutional Animal Care and Use Committee.

### Sampling, sexing, and growth measurement

At the end of experiment, after 180 days, fish were starved for 24 h. The fish were euthanized by tricaine methanesulfonate (MS222) at 250 ppm in water. Then all fish from all tanks were weighed for final weights, and total length was measured to calculate growth performance parameters. All fish were dissected to determine the sex ratios of all replicate tanks via gross examination of the gonad morphology. Single gonad was identified as female and double gonad as male. Meanwhile, four fish from each replicate tanks (3 replicates/group x 4 fish/replicate = 12 fish per group) were randomly selected for blood sampling. Blood was drawn from caudal veins using 3 cc heparinized syringes and transferred into heparinized tubes and kept on crushed ice until centrifugation at 3,600 rpm for 5 min at 4°C to obtain plasma samples. Then separated plasma was transferred into clean centrifuge tubes and stored at -80°C until used for analysis of plasma cortisol level. The same fish were then carefully dissected to obtain samples of liver tissues. These liver tissues were stored in RNAlater (Ambion, USA) and kept at -20°C for the gene expression. All mortality was recorded and survival rate was calculated at the end of the experiment. Ten fish were selected randomly from all tanks (30 per group) for weighing and total length measurement at the beginning and at the ages of 85, 105, 185, and 215 dph at the end of experiment.

### Physiological indices

Gonad, livers, and internal organs were removed and weighed from all fish during sexing. Gonadosomatic index (GSI; gonad Wt/BW x 100), hepatosomatic index (HIS; liver Wt/BW x 100), and viscerosomatic index (VSI; internal organ Wt/BW x 100) were calculated. The plasma cortisol level was assayed according to the manufacture’s protocol for Cortisol Express ELISA Kit (Cayman Chemical™).

### Gene expression

Four fish from each replicate tanks from each group (3 replicates/group x 4 fish/replicate = 12 fish/group) were randomly selected for liver samples and total RNA was isolated from these liver samples using Trizol (Invitrogen, USA™) according to manufacturer’s instructions. The extracted RNA samples were subjected to DNA-free (DNase) treatment to avoid genomic DNA contamination. The quantity of the RNA was evaluated by using Nano-Drop spectrophotometry (Thermo Scientific, USA™). The purity was checked by OD_260_/OD_280_ nm absorption ratio 1.80:2.00. Reverse transcription was performed using a high-capacity cDNA reverse transcription kit (Invitrogen, USA™) following the manufacturer’s instructions for 20 μL total volume of cDNA. Then on the plate, three technical replicates were assigned for each biological replicate (12 fish/group x 3 technical replicates = 36/group). The total volume of 10 RT μL master mix was prepared per reaction on ice by adding 2 μL (10 x RT Buffer), 0.8 μL (25 x dNTP Mix [100 mM]), 2 μL (10 x RT Random Primers), 1 V (RNase Inhibitor), 3.2 μL (Nuclease free H_2_O), and 1 μL (MultiScribe™ Reverse Transcriptase) into a microcentrifuge tube. Then, 10 μL of 25 x RT master mix was added to 10 μL of the RNA sample in each tube and mixed by pipetting and centrifugation in the thermal cycler (Biorad, USA™), which was adjusted following the manufacturer’s instructions, and the resulting cDNA was stored at -20°C. Primer sequence for *Hsp70*, *SOD*, *GPx*, and ß-actin genes are presented in Table 1. Primers were manufactured by IDT (Coralville, IA, USA).

**Table 1:** Primer sequence for the expression study of selected genes in yellow perch, *P*. *flavescens*.

Gene of interest	Forward primer sequence (5’-3’)	Reverse primer sequence (5’-3’)	Function
ß-actin	GCC TCT CTG TCC ACC TTC CA	GGG CCG ACT CAT CGT ACT	Internal standard
*SOD*	GCA TGT AGG AGA CTT GGG CAA T	CCG TGA TTT CTA TCT TGG CAA CA	Oxidative stress
*GPx*	GTC TTG GGT AAC CCC ACC AG	GAC ACT TGG ATG CCA CCT CA	Oxidative stress
*Hsp70*	TGT TGG TCG GTG GCT CAA	TTG AAG AAG TCC TGA AGC AGC TT	Stress protein

PCR amplification was performed using a 7500 Real-Time PCR System (Applied Biosystem®, USA) using 2 μL of cDNA and 18 μL of SYBR select Master Mix (Applied Biosystem, USA), which was prepared by adding 10 μL of 2 x SYBR green Master Mix 6 μL of ddH_2_O, and 1 μL each of forward and reverse primer. The real-time analysis program consisted of one cycle of 95°C for 15 min, and 45 cycles of 95°C for 15s, 56°C for 15s, and 72°C for 10s. On each plate, for every sample, the target gene (gene of interest) and endogenous control (normalize gene: ß-actin) were tested in triplicate. Then expression levels of the genes of interest were normalized to ß-actin, the fluorescence threshold cycle (CT) was determined, and the relative expression of each gene was calculated. The relative expression level of the target gene in the test sample was calculated using the 2^-ΔΔ*CT*^ method [39].

### Statistical analysis

The proportions of males and females in all groups were compared to the hypothetically ideal proportion of 0.5 (1:1 sex ratio in natural population according to Mendelian Genetics and literature) by using Chi-square (X^2^) goodness of fit (P<0.01). The experimental results for the growth parameters, cortisol level, and mRNA expression in each group were statistically analyzed by one-way ANOVA, followed by a post-hoc T-test. A significant difference was considered at *P< 0*.*05*. All data are expressed as the mean ± SD. A multiple regression was run to evaluate the relationship among density, sex weight and length, and a P < 0.05 was considered statistically significant.

## Results

### Sex ratio by density

According to the results shown in Table 2, there were non-significant differences (*P>0*.*01*) in sex ratio of males to females when compared to the expected ratio of 1:1 in normal population in all density groups.

**Table 2:** Percentage of males and females based on macroscopic evaluation of gonad (gonad shape) of yellow perch reared at different stocking densities.

Group (Triplicates)	Observed (N)	Gonad Morphology (%)		*X* ^2^
		Male	Female	
**LD (30/Tank)**	124	50.8	49.2	0.03
**MD (60/Tank)**	192	45.8	54.2	0.71
**HD (120/Tank)**	326	49.4	50.6	0.01

*X*^2^ value is for comparison with an expected normal ratio of males to females of 1:1 or the ideal sex proportion of 0.5 males or females in population. There is no significant difference of all density groups when compared to the normal ratio of males to females *(P*>0.01).

### Sexual size dimorphism in growth by density

**Fig 1:**
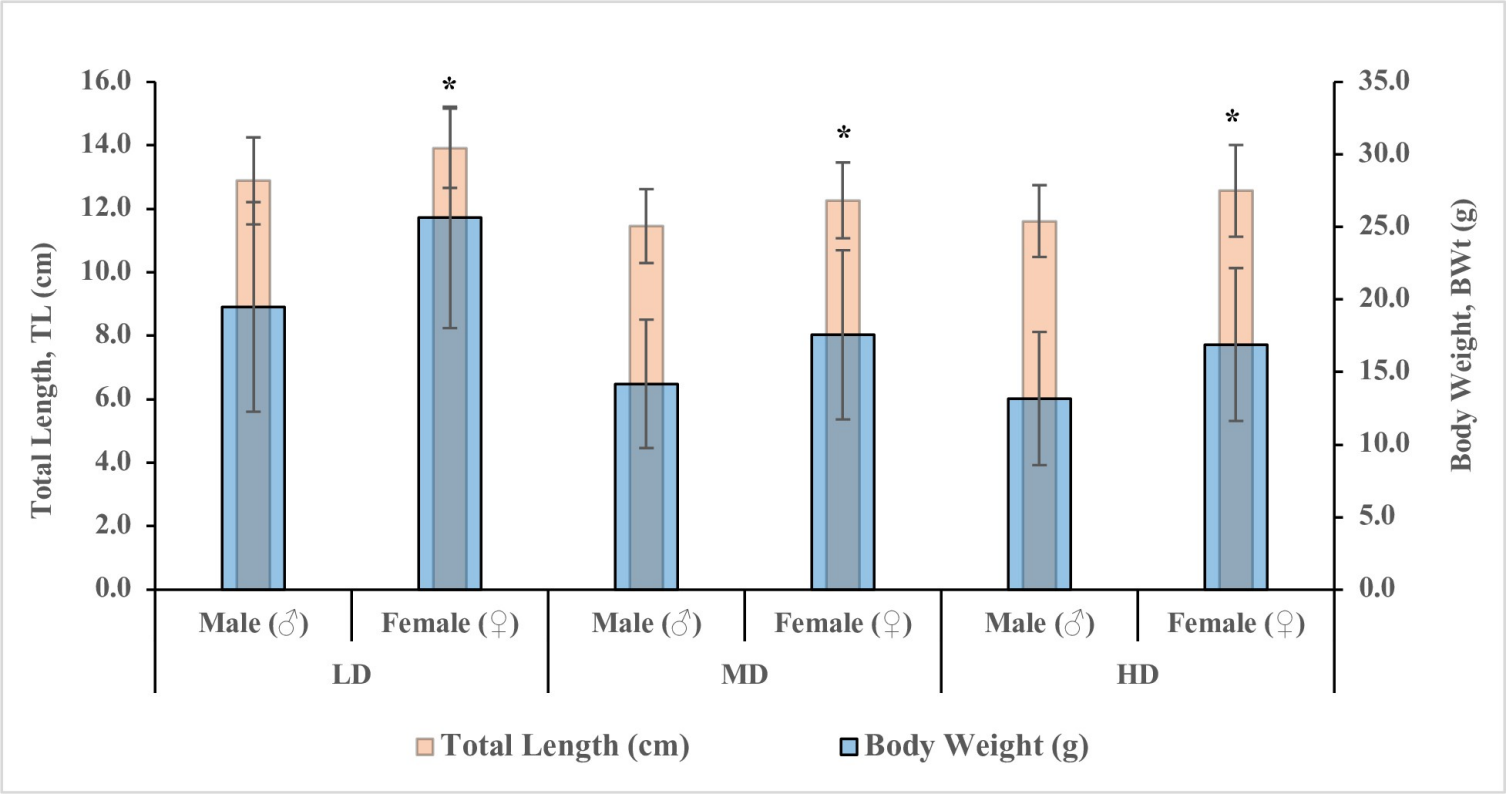
The final total length (cm) and body weight (g) of juvenile male and female yellow perch, *Perca flavescens*, reared at different stocking densities. The data represent the mean ±SD. The mean values within the same group with an asterisk are significantly higher (*P<0*.*05*).

**Fig 2:**
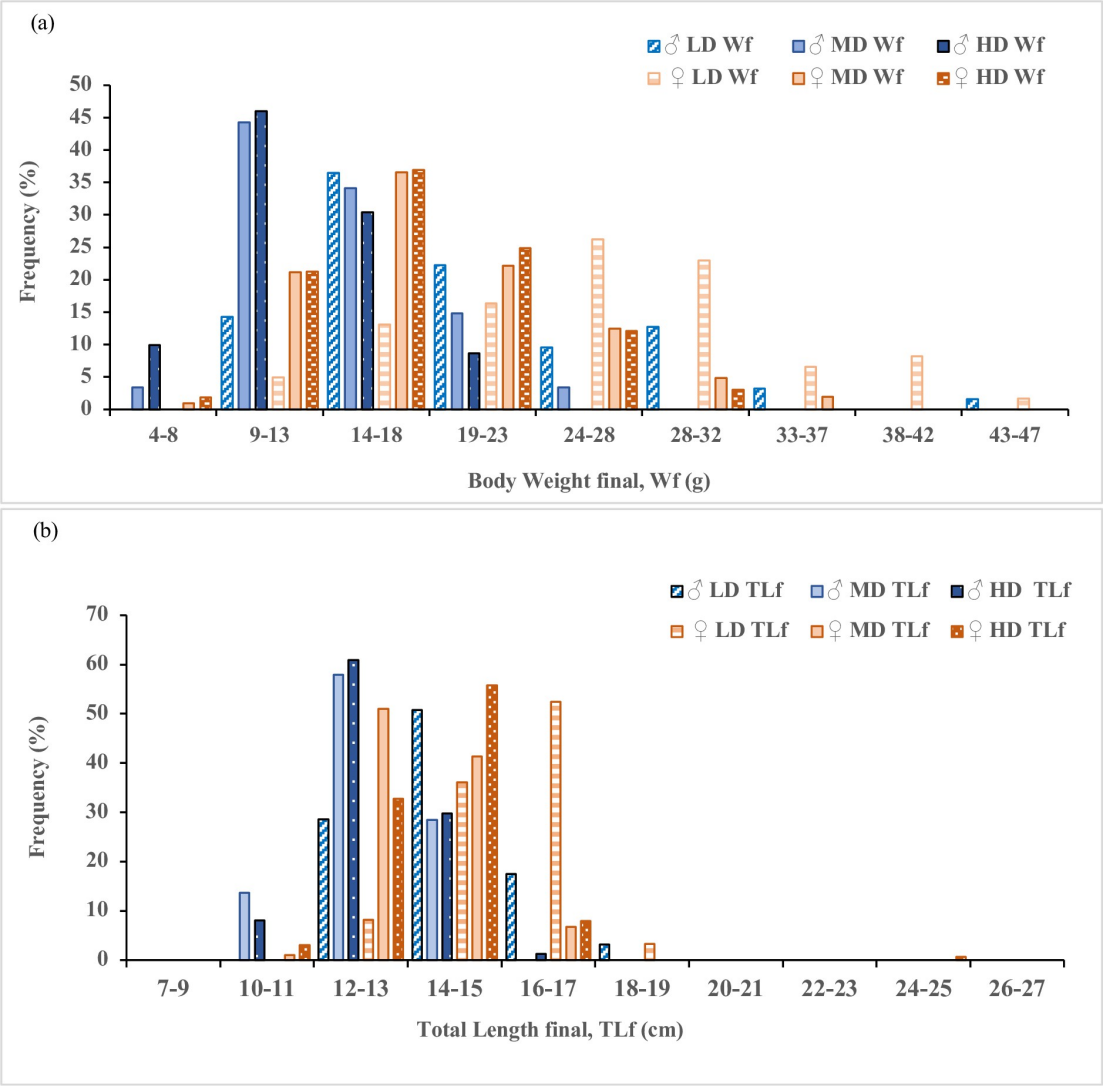
(a) Body weight (g) and total length (cm) frequency (%) distribution of male and female *Perca flavescens* reared at different stocking densities, (b) final Total length (cm) frequency (%) distribution of male and female *Perca flavescens* reared at different stocking densities.

Sexual size dimorphism between sexes for three different stocking densities were evaluated when the gonads/sex could be identified. The results revealed that SSD appeared when male and female were as small as the mean size reaching 11.5 cm and 12.3 cm in total length (TL) or 13.2 g and 16.9 g in body weight (BW), respectively, and female body weight/length were significantly heavier/longer than males in all groups (*P<0*.*05*) (Table 3 and Fig 1). The growth differences between males and females can be obviously observed from their frequency distribution (Fig 2). The specific growth rates (SGR) of females were significantly higher than males (Table 3). Notably, only the LD group females showed significantly higher condition factor (K) (*P<0*.*05*) than males, indicating females in LD grew not only longer, but also fatter with a better state of wellbeing, and SSD was more pronounced in the LD group (Table 3).

**Table 3:** Growth performance for male and female of yellow perch reared at different density.

Group	Final Total Length (cm)		Final weight (g)		Weight gain (g)		SGR (%)		K (%/day)	
	Male (♂)	Female (♀)	Male (♂)	Female (♀)	Male (♂)	Female (♀)	Male (♂)	Female (♀)	Male (♂)	Female (♀)
**LD**	12.88 ± 1.37a	13.91 ± 1.25a*	19.49 ± 7.22^a^	25.66 ± 7.63^a^*	19.46 ± 7.22^a^	25.63 ± 7.63^a^*	3.64 ± 0.21^a^	3.81 ± 0.18^a^*	0.88 ± 0.10^a^	0.93 ± 0.08^a^*
**MD**	11.46 ± 1.16b	12.27 ± 1.20b*	14.19 ± 4.43^b^	17.57 ± 5.83^b^*	14.16 ± 4.43^b^	17.54 ± 5.83^b^*	3.47 ± 0.18^b^	3.59 ± 0.19^b^*	0.88± 0.14^a^	0.89 ± 0.11^b^
**HD**	11.62 ± 1.13b	12.57 ± 1.44b*	13.20 ± 4.58^b^	16.90 ± 5.27^b^*	13.17 ± 4.58^b^	16.87 ± 5.27^b^*	3.43 ± 0.19^b^	3.57 ± 0.18^b^*	0.81 ± 0.08^b^	0.83 ± 0.10^c^

Values are presented as means ±SD. Mean values within the same column with different superscripts are significantly different *(P<0*.*05*). While, mean value between male and female in the same row within the same group followed by asterisk are significantly different (*P<0*.*05*)

The body weight/length of females in LD were significantly heavier/longer than females in MD and HD (*P<0*.*05*) (Fig 1). Similar results were observed for males (*P<0*.*05*) (Table 3). The body weight in both males and females between MD and HD showed no significant difference (*P>0*.*05*; Table 3).

**Fig 3:**
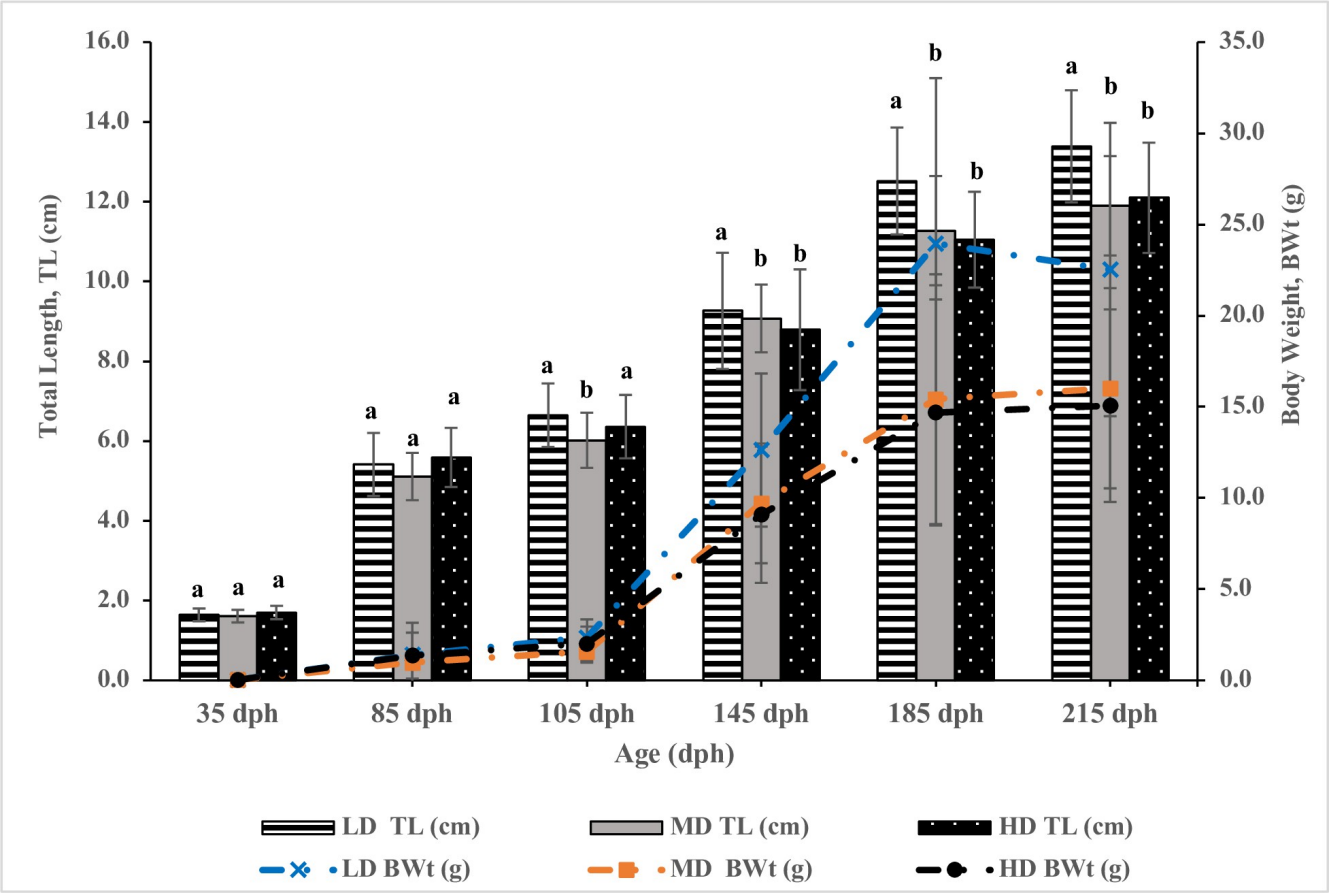
The final total length (cm) and body weight (g) of yellow perch reared at different stocking densities, measured from the beginning at the age of 35 dph until the end of experiment at the age of 215 dph. The data represent the mean ±SD. The mean values within the same column with different superscripts are significantly different (*P<0*.*05*).

When comparing the groups with combined sex data, there was no significant difference detected in the initial body weight among all the density groups (P>0.05) at the beginning of experiment at the age of 35 dph (Fig 3). At the age of 145 dph until the end of experiment at 215 dph, the body weight in LD increased to be significantly higher than MD and HD (P<0.05) (Table 4), but no significant difference was detected between MD and HD (P>0.05) (Fig 3). Similar results were observed for other parameters of final total length, final weight (g), weight gain, SGR, and survival, as shown in Table 4. These results indicated that when density reaches a certain high or limit level, increasing density does not result in further significant effects on growth in both males and females.

**Table 4:** Growth performance and survival rate of yellow perch reared at different density.

Group	Initial weight (g)	Final weight (g)	Weight gain (g)	SGR (%/day)	K (%)	Survival rate (%)
**LD**	0.028 ± 0.007^a^	22.53 ± 8.04^a^	22.50 ± 8.02^a^	3.72 ± 0.22^a^	0.90 ± 0.09^a^	83.91 ± 4.79^a^
**MD**	0.026 ± 0.008^a^	16.02 ± 5.49^b^	15.99 ± 5.49^b^	3.54 ± 0.19^b^	0.92 ± 0.12^a^	75.14 ± 5.40^b^
**HD**	0.027 ± 0.008^a^	15.06 ± 5.28^b^	15.03 ± 5.28^b^	3.50 ± 0.20^b^	0.82 ± 0.09^b^	69.66 ± 4.98^b^

Values are presented as means ±SD. Mean values within the same column with different superscripts are significantly different (*P<0*.*05*)

**Fig 4:**
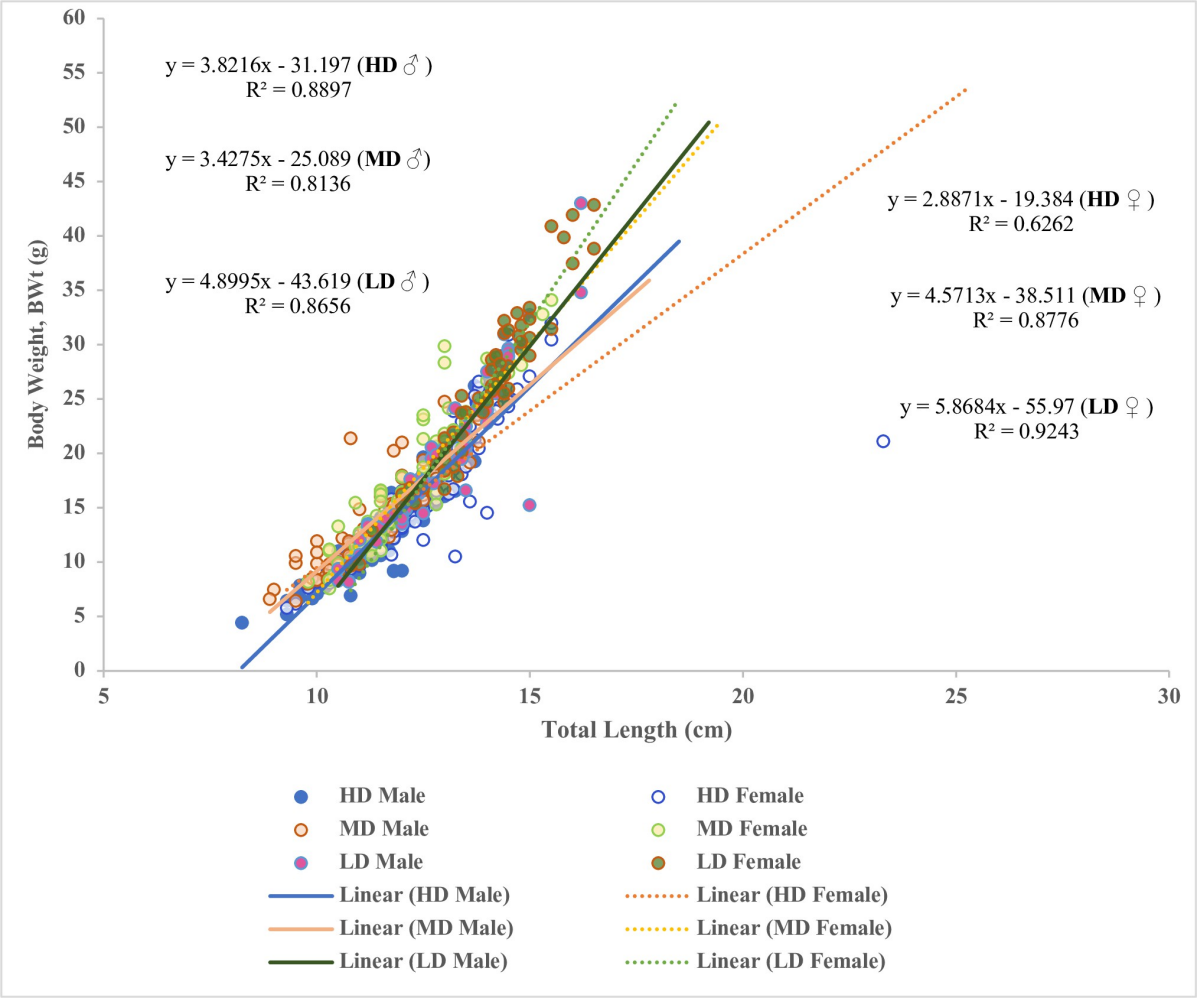
Scaling relationship for final body weight (Wf) to final total length (TLf) for female and male *Perca flavescent* at different densities. The lines represent standard major axis regression slopes for different sexes and different groups. The equations represent regression equations of weight in total length of different sexes and groups.

Final weight (Wf) and final total length (TLf) of both sexes for every group displayed a linear relationship (Fig 4). Females in LD (R^2^ = 0.9243) and MD (R^2^ = 0.8776) displayed significantly stronger correlations than males (R^2^ = 0.8656 and R^2^ = 0.8136); however, in the HD group, males (R^2^ = 0.8897) showed stronger positive correlations than females (R^2^ = 0.6262), indicating HD had a more pronounced effect on females or the fast-growing group (Fig 4). When comparing across the three densities with pooled sex data, R values can be ordered as LD > MD > HD, suggesting HD not only affected growth itself, but also affected normal growth trajectory or body shape in females.

A multiple regression was carried out to examine the relationship among density, sex, BW, and TL of yellow perch. The results of the regression indicated that the four predictors explained a significant amount of 83.6% variance in the value of body weight (F [4,637] = 814.99, p = 0.000, R^2^ = 0.837, R^2^_Adjusted_ = 0.836). The analysis shows that total length (Beta = 3.89, p = 0.000), density (LD: Beta = 2.4, P = 0.000, MD: Beta = 1.71, p = 0.000), and sex (F: Beta = 0.49, P = 0.029) were significant predictors of body weight. The final predictive model was:

$$Body\;weight=-32.21+3.89\left(TL\right)+2.45\left(LD\right)+1.71\left(MD\right)+0.49\;\left(Sex\right)$$

where sex is coded as 1 = Female, 0 = Male and total length is measured in centimeters (cm). YP’s body weight increased 3.89 g for each cm of total length, where on average, LD and MD measured 2.45 cm and 1.71 cm longer than HD, respectively, and females measured 0.49 cm longer than males.

### Sexual size dimorphism in physiological indices by density

The physiological indices of yellow perch are shown in Table 5. Within each density group, females in all three densities showed significantly higher GSI than (*P<0*.*05*) even at the size of ~12 cm. There were no significant differences detected for the GSI when comparing any of the density groups for both males and females (*P>0*.*05*), suggesting the density effect on gonadal development at the size below ~12 cm was minimum. There was no significant difference in HSI between females and males within each group of the three densities (*P>0*.*05*) (Table 5); however, the HSI significantly increased in the fish reared in the LD group when compared to the MD and HD (*P<0*.*05*), suggesting high density affected liver reserves or functions. Comparing VSI between males and females within each density group found that only in LD female was VSI significantly higher than males, and no significant differences (*P>0*.*05*) were detected in VSI in MD and HD groups, indicating high density affected the development of internal organs in fish (Table 5).

**Table 5:** Gonadosomatic index (GSI), Hepatosomatic index (HSI) and Viscerosomatic index (VSI), of yellow perch reared at different stocking densities for 180 days.

Group	GSI (%)		HIS (%)		VSI (%)	
	Ovary (♀)	Testis (♂)	Female (♀)	Male (♂)	Female (♀)	Male (♂)
**LD**	0.43 ± 0.10^a*^	0.09 ± 0.05^a^	3.18 ± 0.75^a^	3.14 ± 0.70^a^	22.88 ± 3.51^a*^	20.85 ± 3.38^a^
**MD**	0.45 ± 0.30^a*^	0.11 ± 0.08^a^	2.89 ± 0.63^b^	2.85 ± 0.66^b^	23.36 ± 4.90^a^	22.48 ± 3.06^b^
**HD**	0.52 ± 0.38^a*^	0.11 ± 0.06^a^	2.86 ± 0.71^b^	2.80 ± 0.60^b^	22.95 ± 6.75^a^	22.06 ± 4.45^ab^

Values are presented as means ±SD. Mean values within the same column with different superscripts are significantly different (*P<0*.*05*). While, mean value between female and male within the same group with asterisk is significantly different *(P<0*.*05*)

#### Plasma cortisol level

**Fig 5:**
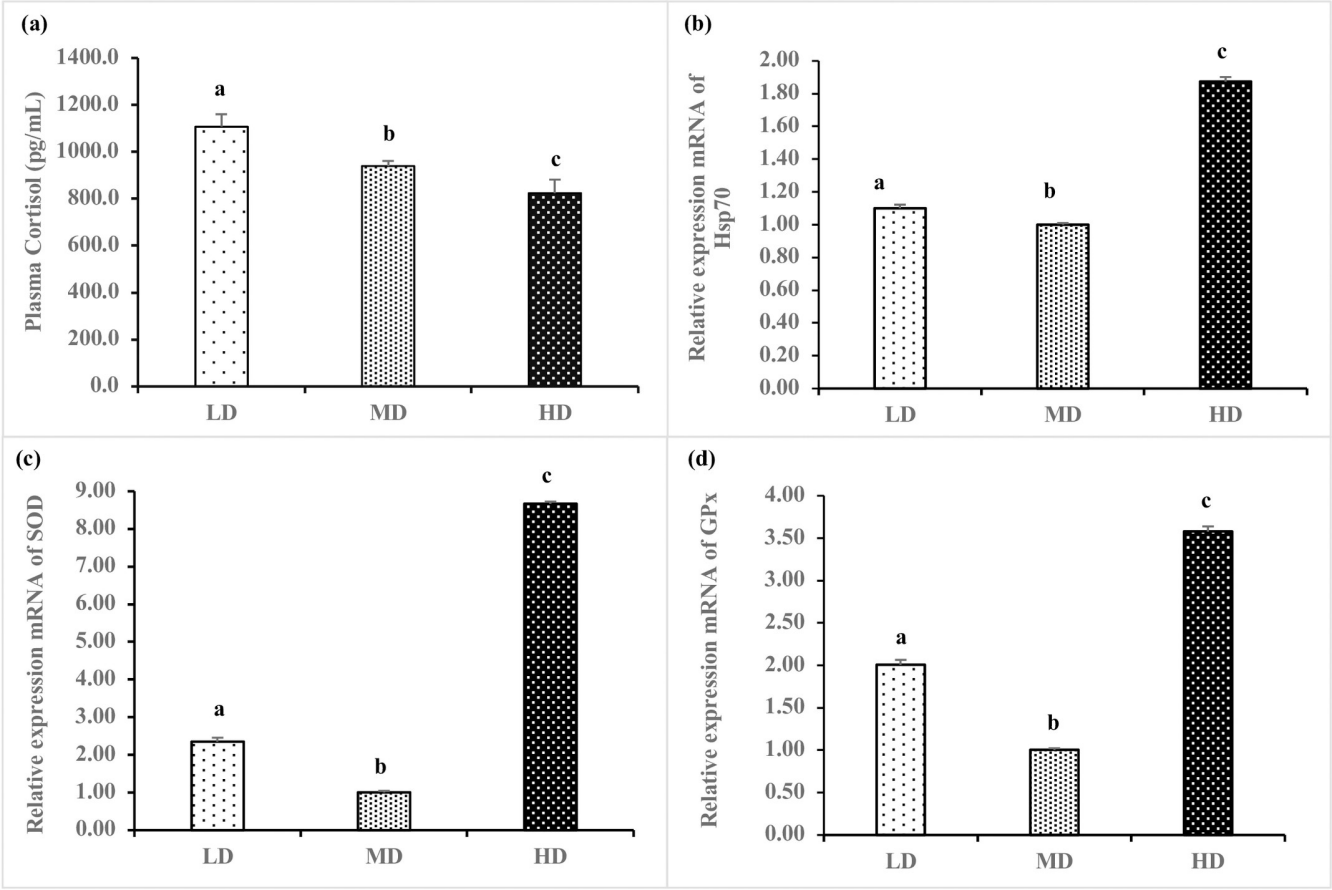
(a) The mean cortisol concentration (pg/mL) of yellow perch reared at different stocking densities, (b) the expression of mRNA *Hsp70* in the liver of yellow perch reared at different stocking densities, (c) the expression of mRNA *SOD* in the liver of yellow perch reared at different stocking densities, and (d) the expression of mRNA *GPx* in the liver of yellow perch reared at different stocking densities. The data represent the mean ±SD. The groups not sharing common letters are significantly different (*P<0*.*05*).

As shown in Fig 5A, the plasma cortisol level significantly decreased with the increasing of stocking density (*P<0*.*05*). The cortisol level was significantly higher (*P<0*.*05*) in the LD group compared to the MD and HD groups. Whereas, the HD group showed significantly low levels (*P<0*.*05*) compared to MD and LD.

### Gene expression

#### The relative expression of mRNA levels of *Hsp70*

The expression of the *Hsp70* gene in liver tissues of yellow perch reared under different stocking densities are presented in Fig 5B. The expression of *Hsp70* in the liver tissue of HD significantly increased (*P<0*.*05*) when compared to the LD and MD groups. Whereas, the *Hsp70* in MD significantly decreased to the lowest level (*P<0*.*05*) among all these groups.

#### The relative expression of mRNA levels of *SOD*

Fig 5C presents the expression of the *SOD* gene in liver tissues of yellow perch reared under different stocking densities. The HD group expressed a significantly higher (*P<0*.*05*) *SOD* level in the liver tissue compared to the LD and MD groups. The expression level of *SOD* in the liver of MD significantly decreased (*P<0*.*05*), and was lowest when compared with the LD and HD groups.

#### The relative expression of mRNA levels of *GPx*

The expression level of *GPx* in liver decreased significantly in the fish reared in MD when compared to HD and LD (*P<0*.*05*), with HD showing the highest increased expression of *GPx* in liver among all groups as shown in Fig 5D.

## Discussion

According to Barcellos et al. [40], stocking density is another external factor that could affect endogenous cortisol levels and, therefore, influence sex determination [41]. The present study indicated that density did not affect the sex ratio during the sexual differentiation stage of yellow perch, although a few experiments on Zebrafish, *Danio rerio*, showed a higher percentage of males in higher stocking densities, which was linked to the cortisol-mediated masculinizing effect [42]. As reported in some other studies, there was no significant evidence of stocking density effects on sex differentiation during the larval period of Rosy Barbs and Dwarf Gouramis [3] or European Sea Bass [43].

Our results showed that SSD occurred when males and females were as small as mean size, reaching 11.5 cm and 12.3 cm in TL or 13.2 g and 16.9 g in BW, respectively, and females were consistently and significantly higher (P<0.05) in various growth parameters compared to males in all groups. Scott el al. [24] reported yellow perch exhibited sexual growth dimorphism at the body length of 11.0 cm. The current study indicated that fish might exhibit SSD earlier than 11.0 cm in body length (BL), since we used mean TL and the smallest fish was around 8.0 cm in TL. Interestingly, our findings showed that, only in LD group, females showed significantly higher condition factor (p<0.05), which indicates a state of better wellbeing, than males. In addition, when comparing pooled sex data across the three densities for analyzing L/W relationships, R values showed LD > MD > HD. The condition factor suggests a general wellbeing and fitness for fish species based on the hypothesis that heavier fish of a given length are in a better physiological condition [44]. These results suggested that HD not only affected growth itself, but also affected normal growth trajectory or body shape and wellbeing of fish, especially females, and SSD was more pronounced in the LD group. Liang et al. [21] and Fontaine et al. [22] also reported female yellow perch exhibit greater growth rate and larger size than males. Nevertheless, this study observed the growth rate of females between groups decreased significantly (P<0.05) with higher densities, as in MD and HD. Hence, females in LD have a greater advantage in this female-biased sexual growth dimorphism.

In the present study, body weight in the LD group was significantly higher than in MD and HD (P<0.05), but no significant differences were detected between MD and HD (P>0.05). Similar results were observed for other parameters of final total length, final weight, weight gain, SGR, and survival, as shown in Table 3. These results indicated that when density reaches a certain limit level, further increasing density would not result in a significant difference on growth of yellow perch. Based on our experiment, stocking 4,000 fry/m^3^ (4 fry/L) would be considered a limit level of density for aquaculture settings in perch.

Stocking density has been identified as an important factor in aquaculture management with the potential to affect fish welfare due to induced stress. Increasing density in fish rearing increases competition for feed and space, which impacts growth performance, behavior, immunity, and metabolism [45]. The negative impact of higher stocking density to growth performance was also observed in snout bream [46], meagre, *Argyrosomus regius* [45], Nile tilapia, *Oreochromis niloticus* [40], chinook salmon, *Oncorhynchus tshawtscha* [47], and bluegill, *Leopomis mar crochius Rafinesque* [48]. Another study showed that increased density impaired feed intake and growth [49], which may be related to oxygen concentrations or water quality [50], whereas the stress response in fish may result in appetite loss and compromised anabolic processes that will reduce growth [51]. This would explain why HD was significantly lower in mean final body weight, weight gain, SGR, and K, which agrees with a previous study where growth performances decreased significantly with increasing stocking density [46] and had a lower survival rate due to chronic stress [3].

High density could provoke crowding stress, subsequently increasing energy requirements and mobilization of energy stores [52]. Therefore, high energy demand under high stocking density causes an increase in mobilization of liver reserves [53], and could be responsible for the significantly lower HSI in the HD group compared to MD and LD in the current study. The finding of low HSI was also observed in common carp (*Crpinus carpio*) reared under high stocking density [54]. It was reported that K and HSI are rough measurements of the energy reserves and nutritional state of fish [55].

Cortisol is one of the most common stress indicators in fish, and it increases during stress [28]. However, it was interesting to find that HD showed significantly lower plasma cortisol levels compared to MD and LD, and this finding is similar to the previous study in juvenile rainbow trout [56]. Nevertheless, according to Santos et al. [57] and van de Nieuwegiessen et al. [58], the value of plasma cortisol was only significantly different when fish were subjected to an acute stress test (netting-stress) and not by chronic stress due to densities treatment [57, 58], as rearing fish in high stocking density is considered a chronic stressor [59]. Therefore, the underlying hypothesis for the high cortisol level in LD in this study could be due to acute stress during sampling rather than the impacts of stocking density. On the other hand, low densities could initiate agonistic behavior and lead to aggression [58, 60, 61].

It was reported that chronic stress situations can lead to the suppression of stress responses caused by a downregulation of the adrenocorticotropic hormone (ACTH) or cortisol receptors [28]. A similar observation was reported when fish housed at high densities failed to show cortisol response after net stress [58]. In addition, another study reported that fish acclimatization, exhaustion of the internal tissues, elevation of cortisol degradation, or decreased influence of stressor contributed to the lack of higher cortisol levels in high-density groups [56, 62, 63]. Hence, the current study would suggest that cortisol level is not a good indicator in fish that were exposed to a chronic stressor. This is because during chronic stress, cortisol levels could be muted over time as the secondary and tertiary stress response mechanisms of the fish take over [28, 30].

Heat shock proteins (*Hsp*) have been studied extensively, are commonly used as an indicator of cellular stress and health status in fish, and thus are referred to as “stress proteins” [51]. In the present study, HD rearing stimulated *Hsp70* in liver that was significantly higher compared to both the LD and MD groups. The increase in *Hsp70* protein expression in larva of gilthead seabream and rainbow trout was described as a mechanism to increase stress tolerance [64]. As observed in sea bass, inducible *Hsp70* in liver was significantly overexpressed only at high rearing density [31]. Whereas in cellular stress response, the *Hsp70* gene plays an important role in maintaining homeostasis in the stress process [65].

Stressor exposure results in the production of reactive oxygen species (ROS), causing an oxidative stress inside the cell [66]. *SOD* and *GPx* are among the first line of the antioxidant defense in fish. Hence, *GPx* genes are considered an accurate estimate of antioxidant capacity and potential biomarkers for fish stress and welfare [67]. The present study showed that yellow perch reared in H-SD had significantly higher expression levels of the anti-oxidation-related genes of *SOD* and *GPx* in liver than those in the LD and MD groups. As observed in the previous study, high density is a source of stress that increased the production of ROS [68]. This is in line with the finding in common carp, *Cyprinus carpio*, reared under high densities that exhibited higher activities in plasma SOD and GPx compared to low-density treatment [69].

## Conclusion

In conclusion, the present study indicated that the stocking density does not have an effect on the sexual determination of yellow perch. However, it was concluded that high density is a chronic stress that has adverse effects on fish wellbeing, SSD, growth, survival, expression of stress-related genes, and mobilization of liver reserve. A stocking density of 1 fish/L or 1,000 fish/m^3^ could be recommended to reduce stress, increase survival, and provide a better state of wellbeing and growth performance for rearing management of juvenile yellow perch and, particularly, a greater advantage for females.

## Supporting information

S1 Dataset (XLSX)
